# Extreme Telomere Length Dimorphism in the Tasmanian Devil and Related Marsupials Suggests Parental Control of Telomere Length

**DOI:** 10.1371/journal.pone.0046195

**Published:** 2012-09-25

**Authors:** Hannah S. Bender, Elizabeth P. Murchison, Hilda A. Pickett, Janine E. Deakin, Margaret A. Strong, Carly Conlan, Daniel A. McMillan, Axel A. Neumann, Carol W. Greider, Gregory J. Hannon, Roger R. Reddel, Jennifer A. Marshall. Graves

**Affiliations:** 1 Research School of Biology, The Australian National University, Canberra, Australian Capital Territory, Australia; 2 Cold Spring Harbor Laboratory, Cold Spring Harbor, New York, United States of America; 3 Cancer Research Unit, Children’s Medical Research Institute, Westmead, New South Wales, Australia; 4 Sydney Medical School, University of Sydney, Sydney, New South Wales, Australia; 5 Department of Molecular Biology and Genetics, Johns Hopkins University School of Medicine, Baltimore, Maryland, United States of America; Tulane University Health Sciences Center, United States of America

## Abstract

Telomeres, specialised structures that protect chromosome ends, play a critical role in preserving chromosome integrity. Telomere dynamics in the Tasmanian devil (*Sarcophilus harrisii*) are of particular interest in light of the emergence of devil facial tumour disease (DFTD), a transmissible malignancy that causes rapid mortality and threatens the species with extinction. We used fluorescent *in situ* hybridisation to investigate telomere length in DFTD cells, in healthy Tasmanian devils and in four closely related marsupial species. Here we report that animals in the Order Dasyuromorphia have chromosomes characterised by striking telomere length dimorphism between homologues. Findings in sex chromosomes suggest that telomere length dimorphism may be regulated by events in the parental germlines. Long telomeres on the Y chromosome imply that telomere lengthening occurs during spermatogenesis, whereas telomere diminution occurs during oogenesis. Although found in several somatic cell tissue types, telomere length dimorphism was not found in DFTD cancer cells, which are characterised by uniformly short telomeres. This is, to our knowledge, the first report of naturally occurring telomere length dimorphism in any species and suggests a novel strategy of telomere length control. Comparative studies in five distantly related marsupials and a monotreme indicate that telomere dimorphism evolved at least 50 million years ago.

## Introduction

Eukaryotic chromosome ends are capped by telomeres, specialised nucleoprotein structures that maintain chromosome stability, limit replicative lifespan, and in doing so, impact carcinogenesis and age-related degenerative disease [1]–[4]. In most cell populations, progressive telomere erosion occurs with each cell division as a result of the end replication problem, until a critically short length is reached and replicative senescence or apoptosis is initiated [5], [6].

Highly proliferative and immortal cells maintain their telomere lengths by activating the enzyme telomerase or by a recombination-based pathway known as Alternative Lengthening of Telomeres (ALT) [7], [8]. Telomerase is composed of two essential components, telomerase RNA (TR), which includes a telomere repeat template region, and telomerase reverse transcriptase (TERT), a catalytic component. In humans, mice and yeast, current models propose that telomerase is limiting, and is preferentially targeted to the shortest telomeres [9]–[11]. Telomerase-positive cancer cell lines typically display short, homogeneous telomere lengths [12]. In contrast, ALT-positive cancer cells are typified by strikingly heterogeneous telomeres and the presence of ALT-associated promyelocytic leukemia protein nuclear bodies (APBs) [13]. Some human neoplasms have both telomerase activity and chromosomes characterised by telomere length heterogeneity suggestive of ALT [14].

The importance of protecting telomere integrity is evident in the diverse spectrum of human disease associated with telomere dysfunction, from neoplasia to pulmonary fibrosis and bone marrow failure [15]–[18]. Dramatic differences in phenotypes associated with telomerase deficiency indicate considerable variation in telomere function between species. Whereas modest telomerase deficiency may result in devastating disease in humans, complete loss of telomerase is tolerated for several generations in laboratory mice, *C.elegans* and plants [19]–[21]. Such interspecies differences are thought to be related to differences in lifespan and reproductive strategies [22]. Long-lived species invest significant energy in cell maintenance and DNA repair in order to successfully propagate germline DNA over longer reproductive intervals. This may provide no selective advantage for short-lived species.

Telomere homeostasis is relatively well understood in humans and model species such as the mouse, *C. elegans* and yeast, yet less is known about telomere regulation in non-model organisms. Here we describe what appears to be a novel cycle of intergenerational, sex-specific telomere lengthening and shortening in the Tasmanian devil and four related species in the marsupial Family Dasyuridae.

The endangered Tasmanian devil *(Sarcophilus harrisii*) is the world’s largest carnivorous marsupial, endemic to the island state of Tasmania. Wild devil populations suffer from critically low genetic diversity, which is thought to have played a role in the recent emergence of a contagious neoplasm known as devil facial tumour disease (DFTD) [23]. DFTD is characterised by unchecked proliferation of a soft tissue neoplasm in the facial subcutis and oral cavity. Affected devils rapidly succumb to starvation due to the locally destructive effects of the tumour, or due to complications associated with thoracic and abdominal metastasis [24], [25].

Efforts to identify the causative agent have demonstrated that DFTD is a stable, immortalised cell line that is propagated when tumour cells are transplanted between devils, probably by biting. This hypothesis is supported by cytogenetic studies [26], [27], microsatellite genotyping [28], mitochondrial DNA sequencing [29], [30], MHC allele typing [28], molecular cytogenetics [31] and whole genome sequencing [30]. Even with significant advances in our understanding of the molecular pathogenesis of DFTD, wild devil numbers have collapsed and at the current rate of decline, extinction of wild devil populations is predicted to occur within 25–35 years [32]. Intensive efforts are therefore underway to elucidate the mechanisms of DFTD transmission and progression, and to translate findings into meaningful conservation strategies.

One of the hallmarks of neoplastic cells is replicative immortality [33]. This requires a telomere length maintenance mechanism such as telomerase activity, which is detected in more than 85% of human neoplasms [34]. The role of telomere length and telomerase activity in DFTD carcinogenesis is therefore of significant interest.

As part of a comparative investigation of telomere length in Tasmanian devil and DFTD chromosomes, we performed quantitative fluorescent *in situ* hybridisation (Q-FISH) on metaphase spreads from healthy devil cells, and cultured DFTD cells. In doing so, we observed a surprising distribution of telomere length within cells from healthy animals. Here we report that the chromosomes of the Tasmanian devil are characterised by unprecedented dimorphism that suggests parental control of telomere length. We propose a model for parental control of telomere length whereby telomere amplification occurs in the male germline and telomere shortening occurs in the female germline. Similar findings in closely related marsupials indicate that telomere length dimorphism evolved at least 50 million years ago and has been conserved across the marsupial Family Dasyuridae. DFTD cells, in contrast, are characterised by uniformly short telomeres and detectable telomerase, consistent with telomerase-mediated telomere length maintenance.

## Results

We characterised telomere length and maintenance mechanisms in healthy male and female Tasmanian devils and in DFTD cells, followed by comparative studies in closely and distantly related marsupial species.

### Telomere Length in the Tasmanian Devil

**Figure 1:**
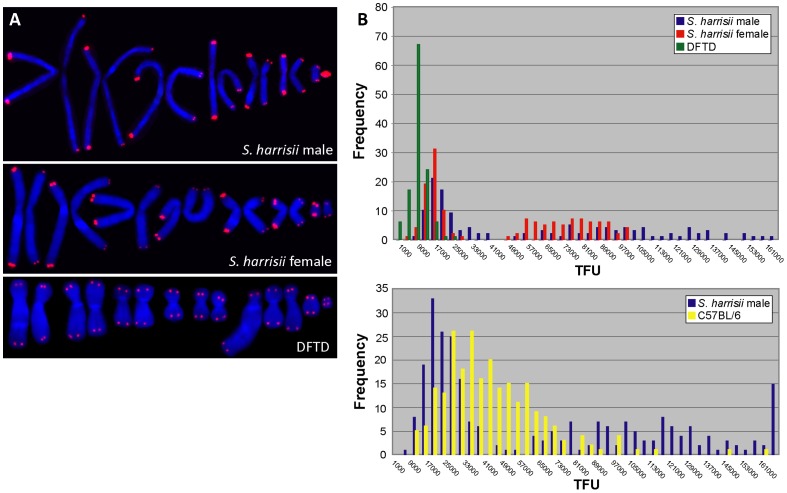
Telomere length dimorphism in the Tasmanian devil. Q-FISH of metaphases from a male and a female Tasmanian devil (lymphocytes and skin fibroblasts, respectively) and a DFTD cell-line using a Cy3-labelled (CCCTAA)_3_ PNA probe. (A) In both male and female Tasmanian devils, homologous chromosomes are characterised by striking differences in telomere length. In male devils, telomeres on the Y chromosome are consistently long and X chromosome telomeres are short. DFTD telomeres are uniformly short. (B) Frequencies of male, female and DFTD telomere fluorescence intensities demonstrate an unusual bimodal distribution of long and short telomeres in male and female devils with marked heterogeneity of the long telomere subset. Telomere lengths are compared to those of C57BL/6 mouse fibroblasts.

The telomere lengths of a male devil and female devil were compared using quantitative fluorescent *in situ* hybridisation (Q-FISH) with a Cy3-labelled (CCCTAA)_3_ peptide nucleic acid (PNA) oligonucleotide. We found that chromosomes in both animals had a striking pattern of long and short telomeres. One homologue from each of the seven chromosome pairs had very long telomeres, whereas the other had considerably shorter telomeres. In images of metaphase chromosomes this correlates with large, bright hybridisation signals on the seven chromosomes with long telomeres. The seven homologues with short telomeres have much smaller, more discrete fluorescence signals (Figure 1A). This result suggested that the two haploid chromosome sets had different telomere lengths.

To test the possibility that these two devils were hybrids between two populations with disparate telomere length, we performed non-quantitative telomere FISH on an additional four animals (2 females, 2 males) from different locations in Tasmania, and on two healthy devils (2 males) from mainland Australian zoos. In all eight animals, haploid chromosome sets were distinguished by strikingly disparate telomere lengths, indicating that telomere length is not a heritable trait segregating in the population. Rather, telomere length dimorphism between homologues is a characteristic feature of Tasmanian devil chromosomes.

The lengths of telomeres on sex chromosomes in male and female devils were examined to investigate the possibility that long and short telomeres were inherited differentially from male and female parents. In all four female devils, X chromosome pairs showed the same striking dimorphism as the autosome pairs. In all four male devils examined, the Y chromosome was characterised by particularly long telomeres, with short telomeres on the X chromosome. This finding is consistent with inheritance of a haploid set of chromosomes with long telomeres from the male parent, and inheritance of a haploid set with short telomeres from the female parent.

Terminal restriction fragment (TRF) analysis of Tasmanian devil telomeres revealed that devil telomeric repeats are probably interspersed with non-repeat sequences (Figure S1), as has been reported for other marsupials [22]. Tasmanian devil telomere length was therefore inferred by comparing hybridisation signals in Q-FISH experiments using a mixture of devil and C57BL/6 mouse fibroblast cell lines (Figure 1B). Comparison of mouse and Tasmanian devil telomere lengths highlighted the dimorphic devil hybridisation signals and demonstrated that short Tasmanian devil telomeres are substantially shorter than the approximately 50 kb long C57BL/6 mouse telomeres. The haploid subset of long devil telomeres is considerably longer than the mouse and is much more heterogeneous than the short telomere subset, with a broader range in telomere fluorescence.

### Telomere Length in DFTD Cells

Q-FISH with the same probe on cultured DFTD cell lines demonstrated that tumour cells were characterised by short, uniform telomeres, with small hybridisation signals on all chromosomes (Figure 1). This was confirmed in an additional four DFTD cell cultures that were prepared from tumour biopsies collected over a period of four years from various locations in Tasmania. Devil facial tumour disease is a clonal somatic cell line that is propagated between host animals [26]–[31], so these results indicate that telomere length has been maintained in the tumour over several generations.

### Telomere Maintenance Mechanisms in the Devil and DFTD

**Figure 2:**
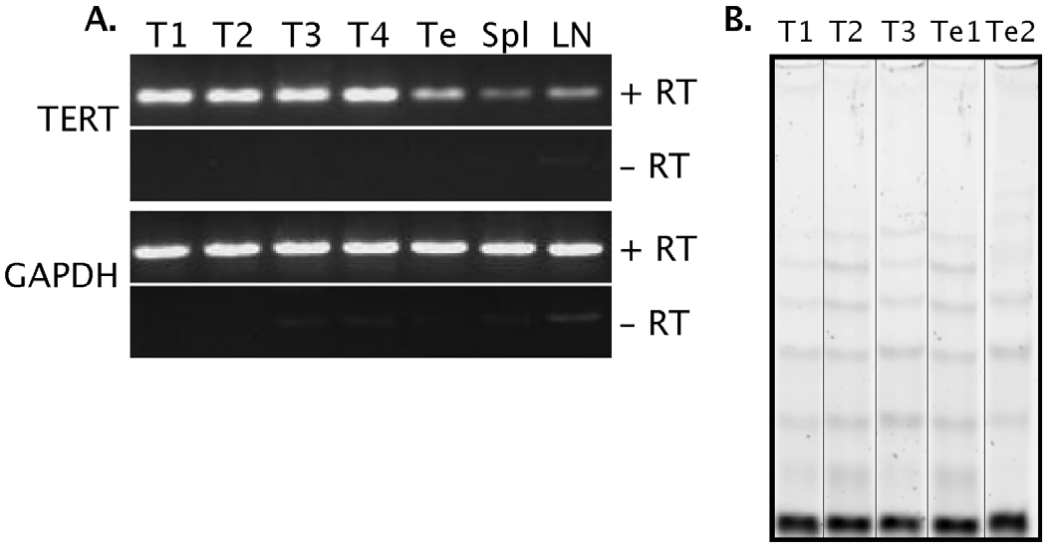
TERT expression and telomerase activity in DFTD and Tasmanian devil tissues. (A) RT-PCR using TERT primers was performed on DNA from Tasmanian devil testis (Te), spleen (Spl) and lymph node (LN) and four independent DFTD tumours (T1–4). GAPDH RT-PCR was performed on DNA from the same tissues as a loading control. RT, reverse transcriptase. (B) Telomerase activity is detected in three DFTD tumors (T1–3) and two testis samples (Te1 and Te2), as measured by TRAP assay.

The presence and activity of telomerase was assessed in samples of various normal Tasmanian devil tissues and in multiple DFTD biopsies. Using semi-quantitative RT-PCR, the catalytic subunit of telomerase, *TERT*, was found to be expressed at low levels in normal devil testis, spleen and lymph node (in descending order of TERT expression; Figure 2A), and was expressed at higher levels in DFTD cells. A non-quantitative telomere repeat amplification protocol (TRAP) assay detected telomerase activity in normal devil testes and in DFTD samples (Figure 2B).

### Telomere Lengths in Other Marsupials

To determine whether telomere length dimorphism is unique to the Tasmanian devil, we examined telomere lengths in animals from other species within the marsupial Family Dasyuridae (Order Dasyuromorphia), which comprises more than 60 species besides the devil. Telomere FISH was performed on the spotted tail quoll (*Dasyurus maculatus*) and three species of dunnart (*Sminthopsis crassicaudata*, *S. macroura* and *S. douglasi*).

**Figure 3:**
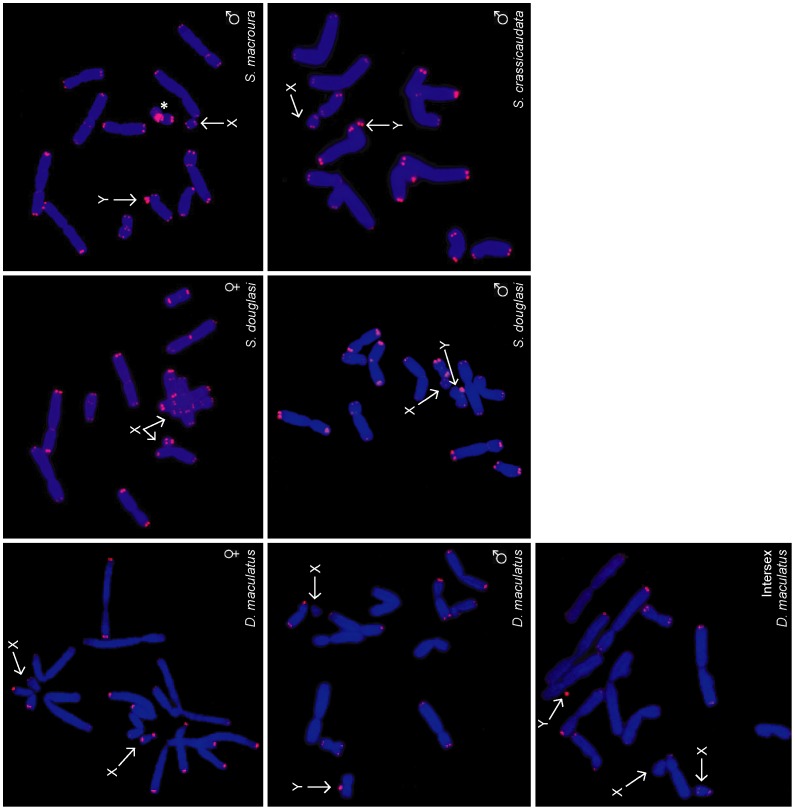
Telomere length dimorphism is a feature of dasyurid chromosomes. FISH demonstrates bimodal telomere length distribution in cells from male, female and intersex spotted tail quolls (*Dasyurus maculatus*), male and female Julia creek dunnarts (*Sminthopsis douglasi*), and male fat-tailed and stripe-faced dunnarts (*S. crassicaudata* and *S. macroura*, respectively). Chromosome 6 of a male stripe-faced dunnart is characterised by interstitial TTAGGG repeats (*).

Like the devil, these species possess markedly dimorphic telomeres, with one haploid set having very long telomeres, and the other having relatively short telomeres. In all male animals, the X chromosome had short telomeres and the Y chromosome had long telomeres (Figure 3). In a wild XXY intersex spotted tail quoll, one X chromosome had short telomeres whereas the second X and the Y chromosome both had long telomeres. Thus, telomere dimorphism is a property of several members of the Family Dasyuridae. We were not able to access animals from the other two families within the marsupial Order Dasyuromorphia.

In a male stripe-faced dunnart (*S. macroura*), the long arm of each chromosome 6 was characterised by interstitial TTAGGG repeats, consistent with previous studies in Sminthopsis [35]. The 6q interstitial repeats were considerably larger on the chromosome with long telomeres; however, additional *S. macroura* were not available to determine whether this observation is due to coincidental association of telomere dimorphism with a randomly segregating polymorphism.

**Figure 4:**
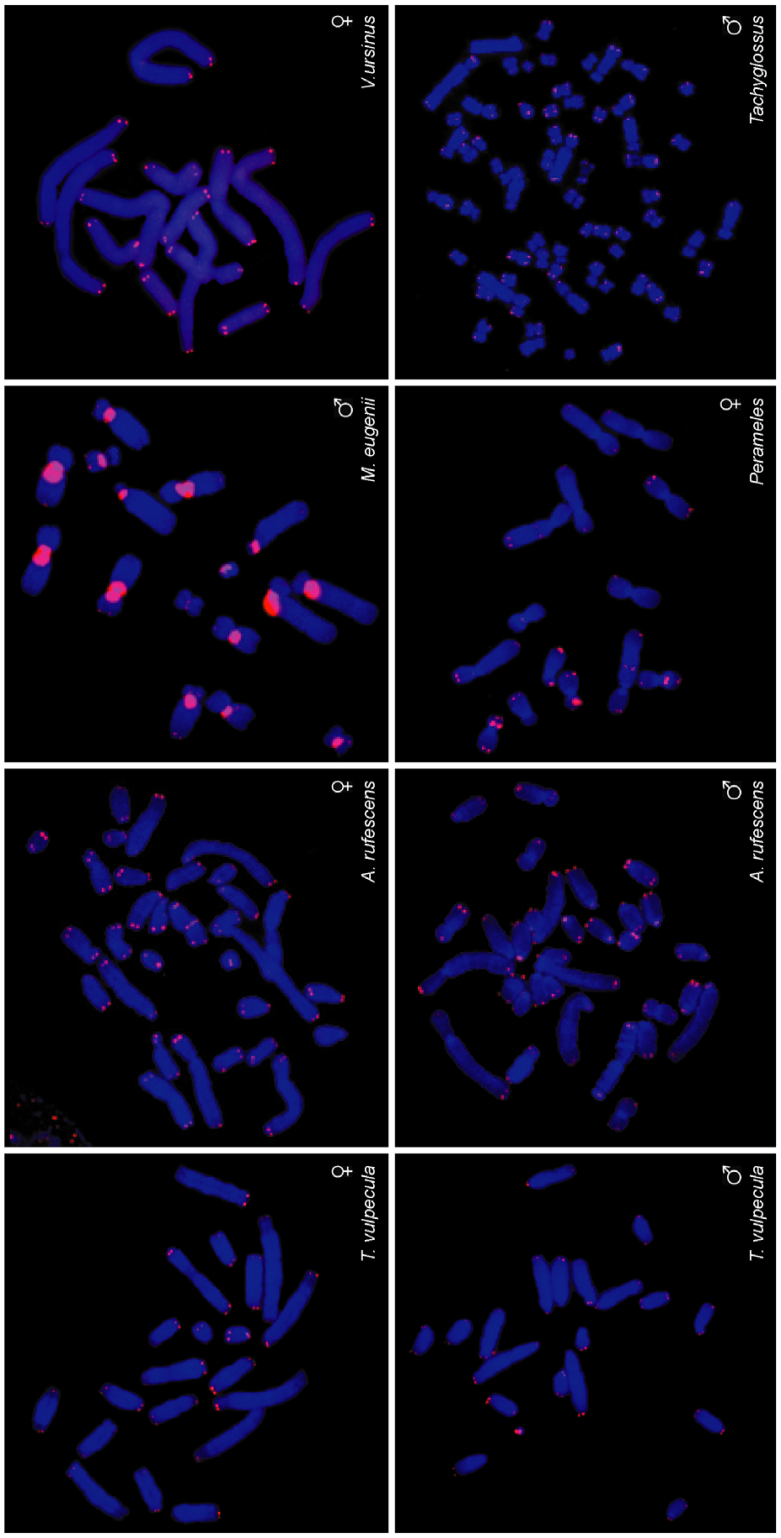
Distantly related marsupials have uniform telomeres. Marsupials in the order Diprotodontia, including the Tammar wallaby (*Macropus eugenii*), the common wombat (*Vombatus ursinus*), the common brushtail possum (*Trichosurus vulpecula*) and the Rufous bettong (*Aepyprymnus rufescens*), and in the order Paramelemorphia, represented by the eastern barred bandicoot (*Perameles gunnii*), have uniform telomeres between homologous chromosomes. The short beaked echidna (*Tachyglossus aculeatus*), a monotreme mammal, likewise has uniform telomeres.

To determine the extent of conservation of telomere dimorphism among marsupials, we compared these results with more distantly related marsupial species of two other Australian orders using FISH (Figure 4). We examined the Tammar wallaby (*Macropus eugenii*), the common wombat (*Vombatus ursinus*), the common brushtail possum (*Trichosurus vulpecula*), the Rufous bettong (*Aepyprymnus rufescens*), representing Order Diprotodontia, and the eastern barred bandicoot (*Perameles gunnii*), representing Order Paramelemorphia.

**Figure 5:**
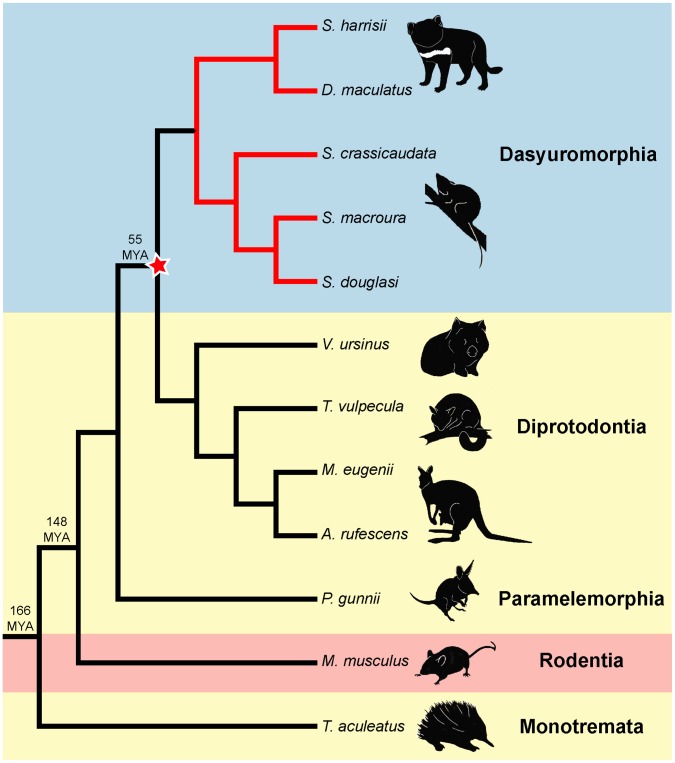
Phylogenetic tree of the species studied here. Telomere length dimorphism is a feature of the Family Dasyuridae and arose after the divergence of the Orders Dasyuromorphia and Paramelemorphia, up to 50 million years ago.

We observed uniform telomeres between homologous chromosomes in all these species. We also investigated telomere length in the short beaked echidna (*Tachyglossus aculeatus*), a monotreme mammal that is basal to the mammalian lineage, and found no dimorphism between homologous chromosomes. Our data suggest that telomere dimorphism is a unique feature of dasyurid marsupials (Figure 5).

## Discussion

We found that chromosomes from healthy Tasmanian devils are distinguished by striking differences in telomere length between homologous chromosomes. This is the first report of naturally occurring telomere length dimorphism in any species and reflects a novel strategy of telomere length control.

### Does Telomere Length Dimorphism Reflect Hybridisation between Two Populations?

An explanation that we considered is that telomere dimorphism is the result of hybridisation between two populations of devils with disparate telomere lengths. A similar phenotype is observed in the F1 hybrids of intercrosses between mice with markedly disparate chromosome lengths [36], [37]. The progeny of *Mus musculus domesticus* and *Mus spretus*, which diverged close to 2 million years ago [38], have bimodal telomere lengths. The short telomeres contributed by *M. spretus* are eventually elongated within somatic cells and the bimodal phenotype is progressively lost with subsequent backcrosses [36], [39]. We tested this hypothesis by examining wild animals from different locations around Tasmania (Figure S2), as well as captive animals from mainland Australian zoos. All animals had dimorphic telomeres, and since it is unlikely that they are all F1 hybrids, we conclude that telomere length dimorphism is a characteristic of normal Tasmanian devils. Similar findings in four closely related dasyurid species indicate that this novel telomere biology is a feature of the marsupial Family Dasyuridae.

### Parent-specific Control of Telomere Length

An intriguing possibility is that telomere length dimorphism is due to a parent-of-origin effect. In humans, telomere length appears to be inherited and strongly influenced by paternal telomere length [40]–[43], and studies of free living sand lizards likewise demonstrates a parent-of-origin effect on telomere length [44].

In the XY dasyurid males in this study, the X chromosome consistently had short telomeres and the Y chromosome had long telomeres. This suggests that the subset of chromosomes with long telomeres is paternally derived in these species. We propose that the dimorphic dasyurid telomere phenotype is due to differential control of telomere length on the paternal and maternal haploid chromosome sets.

How is telomere dimorphism initiated and maintained in dasyurid marsupials? Findings in mice and cattle suggest that telomere length is reset during early embryogenesis, with telomerase-dependent elongation in blastocyst stage embryos [45]. Rather than being due to embryonic resetting, however, we suggest that the telomere dimorphism in Dasyuridae results from differential telomere processing in the male and female of the parental generation with mixing at fertilisation and maintenance through somatic development of the progeny.

### Establishing Telomere Length Dimorphism

**Figure 6:**
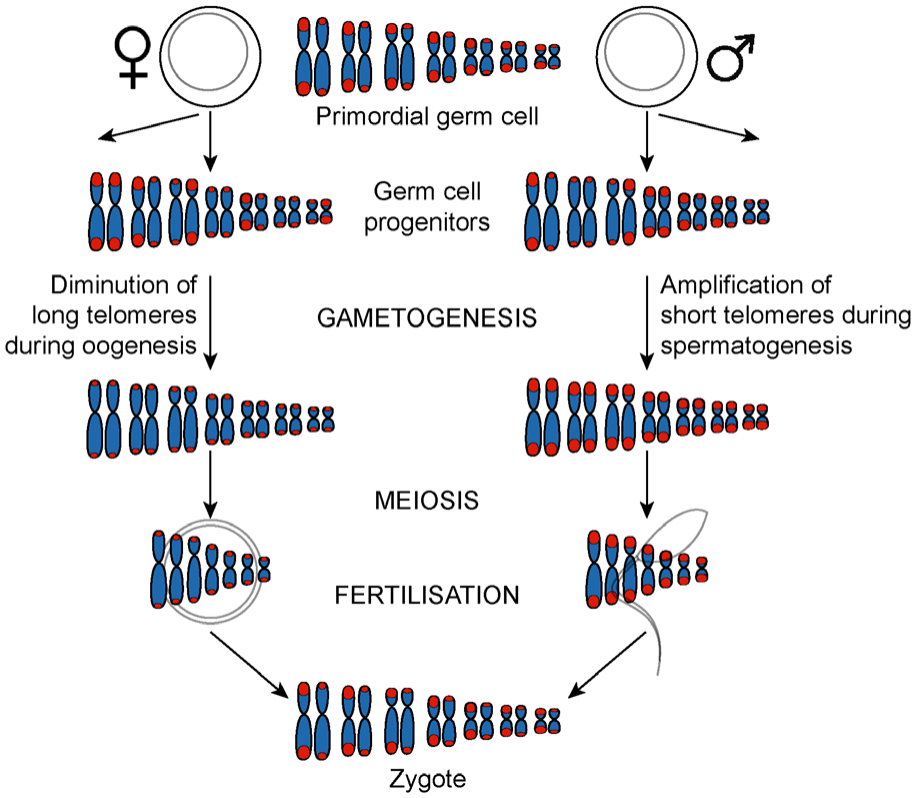
Proposed mechanism of telomere length regulation in the dasyurid germline. Primordial germ cells contain a mixture of long and short telomeres which would be expected to segregate randomly, followed by lengthening and diminution in the paternal and maternal germlines, respectively. Following fertilisation, the zygote contains haploid chromosome sets with long and short telomeres which are maintained in somatic cells.

Establishing dimorphism in an embryo would appear to require both amplification in the male germline and diminution in the female germline, since in both sexes germline progenitor cells must initially have dimorphic telomeres. Long telomeres would have to be shortened in the female germline to produce oocytes with uniformly short telomeres, and short telomeres would have to be lengthened in the male germline to produce sperm with uniformly long telomeres. We therefore present a model in which telomere repeats are amplified in the male germline and diminished in the female germline (Figure 6).

Our observations in an XXY intersex quoll are consistent with this hypothesis. In this animal (with internal testes), one of the X chromosomes had short telomeres and the other long telomeres. As with all male dasyurids, the Y chromosome had long telomeres. Although we have no way of telling whether non-disjunction was maternal or paternal in this wild-caught animal, the simplest explanation is that this XXY quoll received one X chromosome (short telomeres) from the dam and an X and a Y (long telomeres) from the sire, due to telomere elongation in the male germline. This observation also establishes that it is events in the parental generation, and not the sex of the offspring that controls telomere length.

A germline resetting model is also consistent with the finding of uniformly short telomeres in DFTD chromosomes, which have not been subject to germline modifications during continuous passaging *in vivo* for at least 15 years.

A possible alternative to germline resetting is that maternal and paternal chromosome sets are imprinted in the parental germline, so that one haploid set of dasyurid chromosomes experiences accelerated telomere shortening, and the other is subjected to amplification, whose lack of tight control is manifest as wide variation in size. Telomeres on homologous human X chromosomes have been found to shorten at different rates, and epigenetic factors are thought to regulate accelerated attrition of telomeres on the inactive X chromosome [46].

### Maintenance of Telomere Length Dimorphism

How dasyurids develop and maintain two distinct telomere size classes is of great interest. There are four known elements of telomere length control in mammals [47]. There are two telomere shortening mechanisms, namely the gradual telomere attrition which accompanies cellular proliferation and results in part from the end replication problem, and the rapid, regulated telomere shortening known as telomere trimming, and two lengthening mechanisms - telomerase and ALT. TERT is expressed at low levels in normal devil testis, spleen and lymph node, and we found telomerase active in testes, suggestive of telomerase activity in the germline and some somatic tissues (haematopoietic precursors and lymphocytes), consistent with findings in humans [48]. It is therefore possible that the massive lengthening of maternal-origin telomeres that we presume occurs in the male germline may result from sustained, high levels of telomerase activity, most likely unopposed by telomere trimming. The heterogeneous telomere lengths on chromosomes of paternal origin would also be consistent with ALT-mediated lengthening in the male germline. The extent of shortening of paternally-derived telomeres that occurs in the female germline greatly exceeds the shortening that would be expected to occur through gradual replication-associated attrition so it is likely that the rapid telomere trimming mechanism is involved here. The persistence of this dimorphism in somatic cells suggests that the rapid shortening and lengthening mechanisms are mostly inactive in these cells, whereas continued gradual telomere attrition would be compatible with the dimorphism being maintained.

### Evolution of Telomere Length Dimorphism

When did this unusual telomere dimorphism evolve, and what were the conditions that selected for it? The observation of telomere dimorphism in all species of the Family Dasyuridae (Order Dasyuromorphia), but not other marsupial orders, or other mammal groups, provides an estimate of the time that this mechanism evolved. This feature must have been present in the common ancestor at least of dunnarts, quolls and devils, and possibly the common ancestor of the whole Order Dasyuromorphia. The absence of telomere dimorphism in other marsupial orders implies it must have evolved after the divergence of Order Dasyuromorphia and Order Diprotodontia 50 million years ago [49].

What might be the functional consequences or selective advantage of telomere length dimorphism in dasyurids? Little is known about the overall prevalence of neoplasia in dasyurid species, although Tasmanian devils at the San Diego zoo were reported to develop tumours at a higher frequency than other species [50] and it is at least conceivable that this could be associated with aspects of telomere length control in these animals. However, although it is interesting to speculate whether some aspect of the unusual telomere biology of dasyurids predisposes to carcinogenesis, at present we have no evidence for this or that it predisposed to the development of DFTD.

Interestingly, many dasyurids are well known for their semelparous reproductive strategies. The species in this study are iteroparus; however, some *Antechinus* species trade longevity for intense and physiologically costly mating efforts [51] that result in death of males at one year of age due to the systemic effects of markedly elevated testosterone and cortisol levels. Stress has been shown to contribute to telomere attrition [52] and it is interesting to contemplate the significance of telomere lengthening in dasyurid sperm. Perhaps remarkable telomere lengthening during spermatogenesis provides a means to counter the effects of the severe stress males succumb to during mating.

Our findings in the Tasmanian devil and related marsupials suggest a novel system of germline regulation of telomere length. These results provide an exciting avenue for future investigations of the mechanisms that govern parental control of dimorphic telomeres in dasyurid marsupials.

## Materials and Methods

### Ethics Statement

Sample collection was approved by the Australian National University Animal Experimentation Ethics Committee (AEECP R.CG.11.06).

### Animals and Cell Lines

Tasmanian devil tumour, skin and blood samples were obtained from biopsies of live, wild-caught Tasmanian devils (Tables S1 and S2, Figure S2). Animals were trapped for the purposes of disease surveillance and, while under general anaesthesia, were biopsied by a veterinarian. Where euthanasia was indicated for welfare reasons, complete necropsies were performed, allowing more extensive tissue sampling, including testis.

Primary tumour, fibroblast and lymphocyte cultures were initiated following published protocols [26]. Briefly, tumour and skin samples were washed in 10 mL phosphate buffered saline (Invitrogen, Mulgrave, VIC, Australia) with 0.1 mL penicillin-streptomycin solution (Sigma-Aldrich, Castle Hill, NSW, Australia). Cultures were established by disaggregating tissue samples using a scalpel and resuspending cells in 8 mL GIBCO AmnioMAX™-C100 (Invitrogen). Lymphocyte cultures were initiated by isolating the buffy coat and stimulating cells with phytohaemagglutinin (Gibco). Remaining marsupial and monotreme skin fibroblast cell lines were obtained from frozen stocks maintained by the Comparative Genomics Group at the ANU Research School of Biology (Table S3). All cultures were incubated at 35°C in 5% CO_2_.

### Telomere Length Analysis

#### Metaphase preparation

Cell lines were cultured in RPMI 1640 medium with Glutamax, HEPES buffer and 10% foetal bovine serum. Cells were arrested with colcemid (0.1 µg/ml) and harvested 4 hours later by treating with 0.075 M KCl for 20 minutes at 37°C and fixing in chilled methanol/acetic acid (3∶1).

#### Fluorescent *in situ* hybridization

Telomere specific Q-FISH analysis was performed on normal metaphases (fibroblasts and lymphocytes) from two disease-free devils, a tumour cell line and fibroblasts from a C57BL/6 mouse using a Cy3-labelled (CCCTAA)_3_ PNA oligonucleotide as previously described [50]. Chromosomes were counterstained with DAPI and images were acquired using a Zeiss Axioskope microscope and IP-lab Spectrum acquisition software. A minimum of ten metaphases was assessed for each experiment. Cells from an additional four Tasmanian devils, the remaining eight marsupial species and one monotreme were examined by non-quantitative FISH, also using a Cy3-labelled (CCCTAA)_3_ PNA oligonucleotide. These images were captured using Leica DMRXA fluorescence microscope and IP-lab software.

#### Telomere restriction fragment analysis and in-gel hybridization

Agarose plugs containing genomic DNA were prepared from samples of *S. harrisii* spleen and kidney according to kit instructions (Bio-Rad), digested overnight with one or more restriction enzymes (*Mbo*I, *Hinf*I, *Rsa*I, *Msp*I, *Bst*UI, and *Alu*I) and electrophoresed on a 1% agarose gel with 0.5× TBE using a Bio-Rad CHEF MAPPER at 6V/cm using a linear ramped factor, switch times from 12.55 seconds to 1 minute, 8.67 seconds and 120° angle for 28.4 hours at 14°C. After electrophoresis the gel was ethidium bromide stained and dried for 1 hour at 50°C. The dehydrated gel was hybridised to [γ-^32^P]ATP end-labelled oligonucleotide (CCCTAA)_5_, washed and analysed by PhosphorImager (Fuji) as previously described [10].

### Telomerase Activity

#### RNA preparation and RT-PCR

RNA was extracted using standard Trizol procedure from tissues stored in RNAlater. RNA was treated with *DNaseI* and RT-PCR was performed for *TERT* and *GAPDH* using primers TertF 5′-CTGGCAAAAGGTATTCCTGAG-3′, TertR 5′-CAAAACACGTTTAGGGTCCTTG-3′, GapdhF 5′ GACTCAACCACGTATTCGGCTC-3′ and GapdhR 5′- ATATGATTCCACCCATGGCAAGTTCAA-3′. Annealing temperatures were 58°C and 60°C respectively.

#### Telomerase repeat amplification protocol (TRAP)

Telomerase activity was assessed using a commercial telomerase repeat amplification protocol (TRAP) assay (TRAPeze kit, Chemicon). Extracts from tumour and testis samples were prepared following the manufacturer’s instructions. Following PCR amplification of three serial lysate dilutions, PCR products were electrophoresed in a 10% acrylamide gel, stained with ethidium bromide and imaged using a Bio-Rad PharosFX system.

## Supporting Information

Figure S1 **Tasmanian devil telomeres contain non-repeat sequences.** (A) Pulse-field gel electrophoresis and in-gel hybridisation of *Mbo*I digested genomic DNA from Tasmanian devil spleen using an end-labelled (CCCTAA)_3_ probe reveals discontinuous telomere fragments. A C57BL/6 mouse has a single TRF band measuring 25–50 kb. (B) Tasmanian devil kidney DNA samples digested with *Mbo*I (1), *Hinf*I and *Rsa*I (2), *Msp*I, *Bst*UI, *Alu*I (3) and *Hinf*I, *Rsa*I, *Msp*I, *Bst*UI, *Alu*I (4) produce fragments of varying sizes.
(PDF)

Figure S2 **Trapping locations in Tasmania.** Tasmanian devil and tumour samples were collected from various sites in Tasmania. Samples were also obtained from two mainland zoos.
(PDF)

Table S1 **Tasmanian devil (** ***Sarcophilus harrisii*** **) fibroblast and lymphocyte preparations.**
(DOCX)

Table S2 **Devil facial tumour disease samples.**
(DOCX)

Table S3 **Marsupial and monotreme cell lines.**
(DOCX)
