# Supplementary figures and images for: Extreme Telomere Length Dimorphism in the Tasmanian Devil and Related Marsupials Suggests Parental Control of Telomere Length
Source: PLoS One. 2012 Sep 25;7(9):e46195. doi: 10.1371/journal.pone.0046195 (PMC3458001; doi:10.1371/journal.pone.0046195)

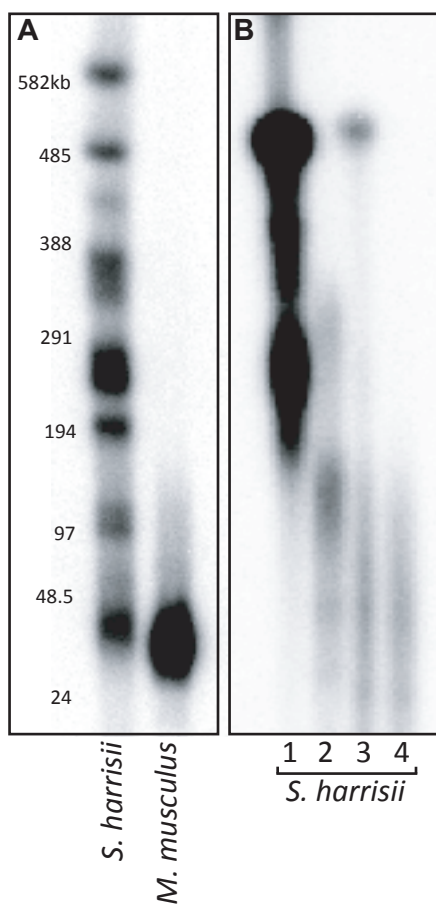

Supplement: Figure S1 — Tasmanian devil telomeres contain non-repeat sequences. (A) Pulse-field gel electrophoresis and in-gel hybridisation of MboI digested genomic DNA from Tasmanian devil spleen using an end-labelled (CCCTAA)3 probe reveals discontinuous telomere fragments. A C57BL/6 mouse has a single TRF band measuring 25–50 kb. (B) Tasmanian devil kidney DNA samples digested with MboI (1), HinfI and RsaI (2), MspI, BstUI, AluI (3) and HinfI, RsaI, MspI, BstUI, AluI (4) produce fragments of varying sizes. (PDF) [file pone.0046195.s001.pdf]

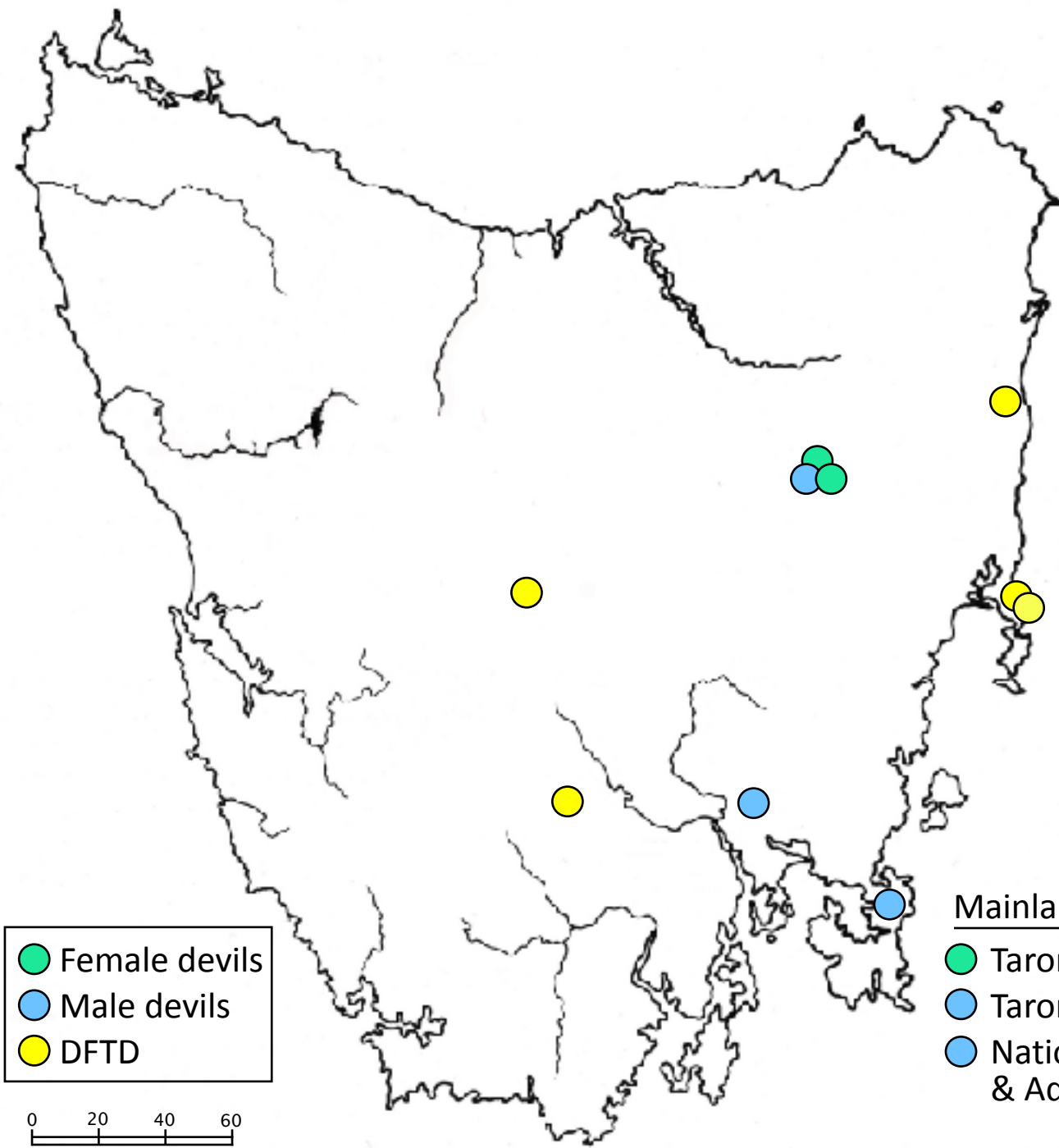

● Female devils  
● Male devils  
● DFTD

Mainland zoos:  
● Taronga Zoo  
● Taronga Zoo  
● National Zoo & Aquarium

0 20 40 60  
Km

Supplement: Figure S2 — Trapping locations in Tasmania. Tasmanian devil and tumour samples were collected from various sites in Tasmania. Samples were also obtained from two mainland zoos. (PDF) [file pone.0046195.s002.pdf]
