# Supplementary material for: Extreme Telomere Length Dimorphism in the Tasmanian Devil and Related Marsupials Suggests Parental Control of Telomere Length
Source: PLoS One. 2012 Sep 25;7(9):e46195. doi: 10.1371/journal.pone.0046195 (PMC3458001; doi:10.1371/journal.pone.0046195)
Supplement: Table S1 — Tasmanian devil ( Sarcophilus harrisii ) fibroblast and lymphocyte preparations. (DOCX) [file pone.0046195.s003.docx]

**Table S1**

Tasmanian devil (*Sarcophilus harrisii*) fibroblast and lymphocyte preparations

| Accession number | Age | Sex | Health status | Year collected | Location | Tissue cultured | Passages through culture |
| --- | --- | --- | --- | --- | --- | --- | --- |
| 06.1565 | 1yr | Female | Healthy | 2006 | Epping Forest, TAS^a^ | Peripheral blood | 0 |
| 06.1657 | 4yr | Female | Healthy | 2006 | Epping Forest, TAS | Peripheral blood | 0 |
| N/A | Unknown | Female | Healthy | 2006 | Taronga Zoo, NSW^b^ | Skin fibroblasts | <10 |
| 06.1196 | Unknown | Male | DFTD | 2006 | Tea Tree, TAS | Peripheral blood | 0 |
| 06.1561 | 1yr | Male | Unknown | 2006 | Forestier Peninsula, TAS | Peripheral blood | 0 |
| 06.1568 | 2yr | Male | Healthy | 2006 | Epping Forest, TAS | Peripheral blood | 0 |
| N/A | Unknown | Male | Euthanased, DFTD free | 2008 | National Zoo and Aquarium, ACT^c^ | Skin fibroblasts | <10 |
| N/A | Unknown | Male | Healthy | 2006 | Taronga Zoo, NSW | Skin fibroblasts | <10 |

^a^ Tasmania (TAS)

^b^ New South Wales (NSW)

^c^ Australian Capital Territory (ACT)
