# Supplementary material for: Extreme Telomere Length Dimorphism in the Tasmanian Devil and Related Marsupials Suggests Parental Control of Telomere Length
Source: PLoS One. 2012 Sep 25;7(9):e46195. doi: 10.1371/journal.pone.0046195 (PMC3458001; doi:10.1371/journal.pone.0046195)
Supplement: Table S2 — Devil facial tumour disease samples. (DOCX) [file pone.0046195.s004.docx]

**Table S2**

Devil facial tumour disease samples

| Accession number | Age | Sex | DFTD strain | Year collected | Location^a^ | Passages through culture |
| --- | --- | --- | --- | --- | --- | --- |
| 04.3089 | 3yr | Female | 1 | 2004 | Bronte | <20 |
| 05.2569 | 3yr | Male | 2 | 2005 | Fentonbury | <10 |
| 06.2772 | 1yr | Male | 1 | 2006 | St Mary’s | <10 |
| 07.0306 | Unknown | Male | 3 | 2007 | Coles Bay | <10 |
| 07.0192 | 2yr | Male | 4 | 2007 | Coles Bay | <10 |

^a^All locations are within Tasmania
