# Supplementary material for: Extreme Telomere Length Dimorphism in the Tasmanian Devil and Related Marsupials Suggests Parental Control of Telomere Length
Source: PLoS One. 2012 Sep 25;7(9):e46195. doi: 10.1371/journal.pone.0046195 (PMC3458001; doi:10.1371/journal.pone.0046195)
Supplement: Table S3 — Marsupial and monotreme cell lines. (DOCX) [file pone.0046195.s005.docx]

**Table S3**

Marsupial and monotreme cell lines

| **Species** | **Sex** | **Passage number** |
| --- | --- | --- |
| Spotted tail quoll  *(Dasyurus maculatus)* | F | 3 |
|  | M | 2 |
|  | Intersex | 3 |
| Stripe-faced dunnart  *(Sminthopsis macroura)* | M | 10 |
| Julia Creek dunnart  *(Sminthopsis douglasi)* | F | 6 |
|  | M | 8 |
| Fat-tailed dunnart  *(Sminthopsis crassicaudata)* | M | 8 |
| Brushtail possum  (*Trichosurus vulpecula*) | F | 57 |
|  | M | 4 |
| Rufous bettong  (*Aepyprymnus rufescens*) | F | 8 |
|  | M | 9 |
| Tammar wallaby  (*Macropus eugenii*) | M | 8 |
| Common wombat  (*Vombatus ursinus*) | F | 7 |
| Eastern barred bandicoot  (*Parameles gunnii*) | M | Information unavailable |
| Short-beaked echidna  (*Tachyglossus aculeatus*) | M | 8 |
